# Cytotoxic activities of phytochemicals from *Ferula* species

**DOI:** 10.1186/2008-2231-21-39

**Published:** 2013-05-23

**Authors:** Seied Mojtaba Valiahdi, Mehrdad Iranshahi, Amirhossein Sahebkar

**Affiliations:** 1 Institute of Inorganic Chemistry, University of Vienna, Vienna, Austria; 2 Biotechnology Research Center and School of Pharmacy, Mashhad University of Medical Sciences, 91775-1365, Mashhad, Iran; 3 Department of Modern Sciences and Technologies, Faculty of Medicine, Mashhad University of Medical Sciences, Mashhad, Iran

**Keywords:** Cancer, Cytotoxicity, Phytochemical, Ferula

## Abstract

**Background:**

*Ferula* species are reputed in folk medicine for the treatment of a variety of disorders. There have been sporadic reports on the chemopreventive and chemosensitizing activities of some terpenoid coumarin derivatives from the genus *Ferula*. The present study investigated the cytotoxic activity of 11 phytochemicals (conferone, farnesiferol A, acantrifoside E, mogoltadone, diversin, galbanic acid, herniarin, 7-isopentenyloxycoumarin, umbelliprenin, stylosin and tschimgine) from *Ferula* species together with a newly synthesized prenylated derivative of curcumin (gercumin II).

**Methods:**

Cytotoxic activity of phytochemicals was evaluated against ovarian carcinoma (CH1), lung cancer (A549) and melanoma (SK-MEL-28) cell lines using MTT assay.

**Results and conclusion:**

Overall, moderate cytotoxic activity was observed from the tested compounds with IC_50_ values in the micromolar range. The highest activity against CH1 and A549 lines was from conferone while stylosin and tschimgine were the most potent compounds against SK-MEL-28 line. In conclusion, the findings of the present investigation did not support a potent cytotoxic activity of the tested phytochemicals against CH1, A549 and SK-MEL-28 cell lines. With respect to previous reports, the beneficial impact of these phytochemicals in cancer therapy may be more attributable to their chemopreventive or chemosensitizing activity rather than direct cytotoxic effects.

## Introduction

During recent decades, there has been an increasing demand for finding newer and safer chemotherapeutic agents. Natural products, in particular plants, have long been used for their medicinal properties in several traditional systems of medicine. Over 60% of current cytotoxic agents have been derived from natural sources including plants, marine organisms and microorganisms, either directly or by chemical synthesis based on natural lead compounds [1,2]. Therefore, natural products have a wide application in cancer chemotherapy [2].

Genus *Ferula* (Apiaceae) comprises about 170 species, of which 30 have been included in Iranian flora and some are endemic. Plants belonging to this genus are distributed throughout central Asia, Mediterranean region and Northern Africa, and are well reputed in traditional medicine for the treatment of a variety of disorders [3]. To date, more than 70 *Ferula* species have been subjected to phytochemical analysis, and findings have led to the identification of this genus as a good source of bioactive compounds including terpenoid derivatives [4-7]. In the present work, we sought to determine the cytotoxic activity of phytochemicals isolated from *Ferula* species, and also a novel synthetic derivative of curcumin, against tumor cell lines originating from melanoma, ovarian and lung carcinoma.

## Materials and methods

### Test compounds

**Figure 1:**
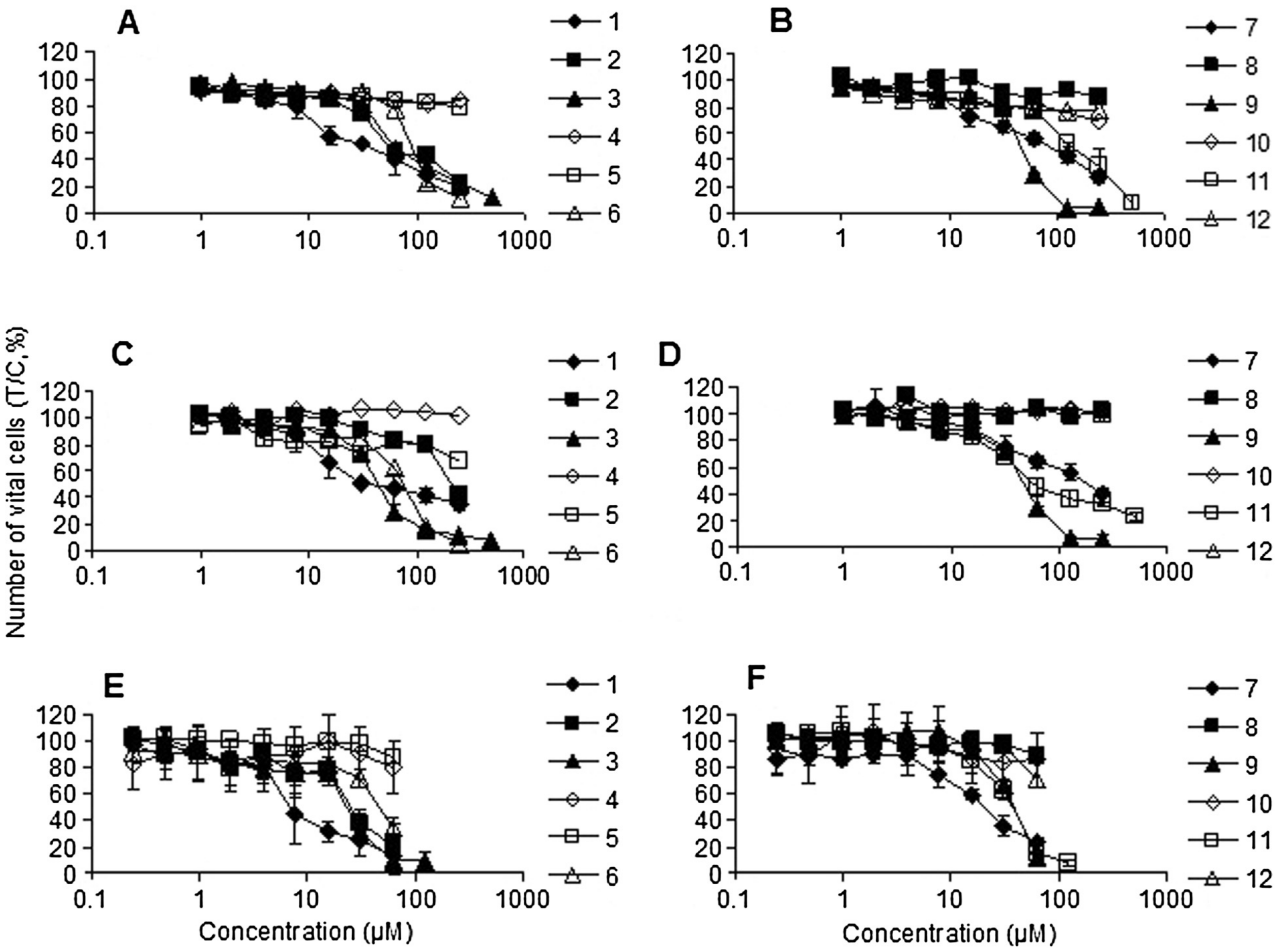
**Concentration-effect curves of tested phytochemicals in A549 (A, B), SK-MEL-28 (C, D) and CH1 cells (E, F), obtained by the MTT assay (96 h exposure).** 1: Conferone; 2: farnesiferol A; 3: stylosin, 4: diversin; 5: herniarin; 6: galbanic acid; 7: mogoltadone; 8: 7-isopentenyloxycoumarin; 9: tschimgine; 10: acantrifoside E and 11: umbelliprenin; 12: gercumin II.

Chemical structures of test compounds are shown in Figure 1. 7-prenyloxycoumarins namely umbelliprenin, 7-isopentenyloxycoumarin and herniarin were chemically synthesized as described previously [8]. Briefly, synthesis was performed by reaction between 7-hydroxycoumarin (1 M) and relevant prenyl bromides (1.5 M) in acetone at room temperature, and in the presence of DBU (1, 8-diazabicyclo [5.4.0] undec-7-ene) (2 M). After 24 hrs, the mixture was concentrated under reduced pressure. The products were purified by column chromatography and their structures were characterized using ^1^H- and ^13^C-NMR (Additional files 1, 2 and 3).

Monoterpene esters, stylosin and tschimgine, were isolated from *F*. *ovina* root extract. In brief, powdered roots of *F*. *ovina* (500 g) were extracted by dichloromethane (3 L) using maceration method (36 h), yielding a residue (93 g). Part of the extract (21 g) was subjected to column chromatography on silica gel (5 × 60 cm) using petroleum ether/ethyl acetate (20/1) as an initial solvent with gradual increasing of solvent polarity up to 100% ethyl acetate. Stylosin (706 mg; mp: 160-162°C) and tschimgine (1691 mg; mp: 158-159°C) were obtained as pure solid crystals from the column and their structures were confirmed by comparison of ^1^H- and ^13^C-NMR spectra as well as melting point value with those of a previous report [9] (Additional files 4, 5, 6, 7 and 8).

Galbanic acid (Additional file 9), farnesiferol A (Additional file 10), diversin (Additional file 11), conferone (Additional file 12), acantrifoside E (Additional files 13, 14 and 15) and mogoltadone (Additional file 16) were isolated from the roots of *F*. *szowitsiana*[6], *F*. *persica*[10], *F*. *diversivittata*[11], *F*. *flabelliloba*[12], *F*. *gummosa*[13] and *F*. *persica*[14], respectively.

For the synthesis of gercumin II, curcumin (2 mmol ~ 736 mg) was dissolved in acetone (50 cc) and reacted with geranyl bromide (2 mmol ~ 400 μL) in the presence of DBU (2 mmol ~ 300 μL). Then, the mixture was refluxed for 150 minutes at 37°C. After the reaction was completed (confirmed by TLC), the products were isolated by decantation of the reaction mixture with distilled water (75 cc) and ethyl acetate (75 cc). After separation and solvent evaporation of the organic phase, the products were purified by Silica Gel column chromatography with petroleum ether/acetone/methanol (5/1/0.1 v/v) system as eluent. The fractions (80 mL each) were compared by TLC [petroleum ether/acetone/methanol (2.5/1/0.1 v/v)], and those giving similar spots were combined, solvent evaporated and crystallized. Gercumin II was obtained as yellow crystals (mp: 62-64 C; yield: 38%). The chemical structure of gercumin II was confirmed by ^1^H- and ^13^C-NMR experiments (Additional file 17).

#### Cell lines and culture conditions

CH1 (ovarian carcinoma, human) cells were donated by Lloyd R. Kelland (CRC Centre for Cancer Therapeutics, Institute of Cancer Research, Sutton, UK). A549 (non-small cell lung cancer, human) and SK-MEL-28 (malignant melanoma, human) cells were kindly provided by Brigitte Marian (Institute of Cancer Research, Department of Medicine I, Medical University of Vienna, Austria) and Christoph Hoeller (Dermatology Clinic, Medical University of Vienna, Austria). Cells were grown in 75 cm^2^ culture flasks (Iwaki/Asahi Technoglass) as adherent monolayer cultures in Minimal Essential Medium (MEM) supplemented with 10% heat-inactivated fetal bovine serum, 1 mM sodium pyruvate, and 2 mM L-glutamine (all purchased from Sigma-Aldrich) without antibiotics. Cultures were maintained at 37°C in a humidified atmosphere containing 5% CO_2_ and 95% air.

#### Cytotoxic activity tests in cancer cell lines

Cytotoxic activity in the cell lines mentioned above was determined by the colorimetric MTT assay (MTT = 3-(4,5-dimethyl-2-thiazolyl)-2,5-diphenyl-2H-tetrazolium bromide, purchased from Fluka). Cells were harvested from culture flasks by trypsinization and seeded in 100 μL aliquots in MEM supplemented with 10% heat-inactivated fetal bovine serum, 1 mM sodium pyruvate, 2 mML-glutamine, and 1% non-essential amino acids (100x) into 96-well microculture plates (Iwaki/Asahi Technoglass). Cell densities per well were adjusted to 1.5 × 10^3^ (CH1), 4.0 × 10^3^ (A549), and 4.0 × 10^3^ (SK-MEL-28), to ensure exponential growth of untreated cells. Cells were allowed to settle and resume exponential growth in drug-free complete culture medium for 24 h, followed by addition of dilutions of the test compounds in 100 μL/well of the same medium. Test compounds were used from fresh stock solutions in dimethylsulfoxide (DMSO) and diluted in media or buffer as appropriate to a maximum of 0.3% DMSO. After continuous exposure for 96 h, the medium was replaced by 100 μL/well RPMI 1640 medium (supplemented with 10% heat-inactivated fetal bovine serum and 4 mML-glutamine) plus 20 μL/well solution of MTT in phosphate-buffered saline (5 mg/mL) (all purchased from Sigma-Aldrich). After incubation for 4 h, medium/MTT mixtures were removed, and the formazan product formed by viable cells was dissolved in DMSO (150 μL/well). Optical densities were measured at 550 nm with a microplate reader (Tecan Spectra Classic), using a reference wavelength of 690 nm to correct for non-specific absorption. The quantity of viable cells was expressed as percentage of untreated controls, and 50% inhibitory concentrations (IC_50_) were calculated from concentration-effect curves by interpolation. Evaluation was based on means from three independent experiments, each comprising six replicates per concentration level.

## Results and discussion

**Figure 2:**
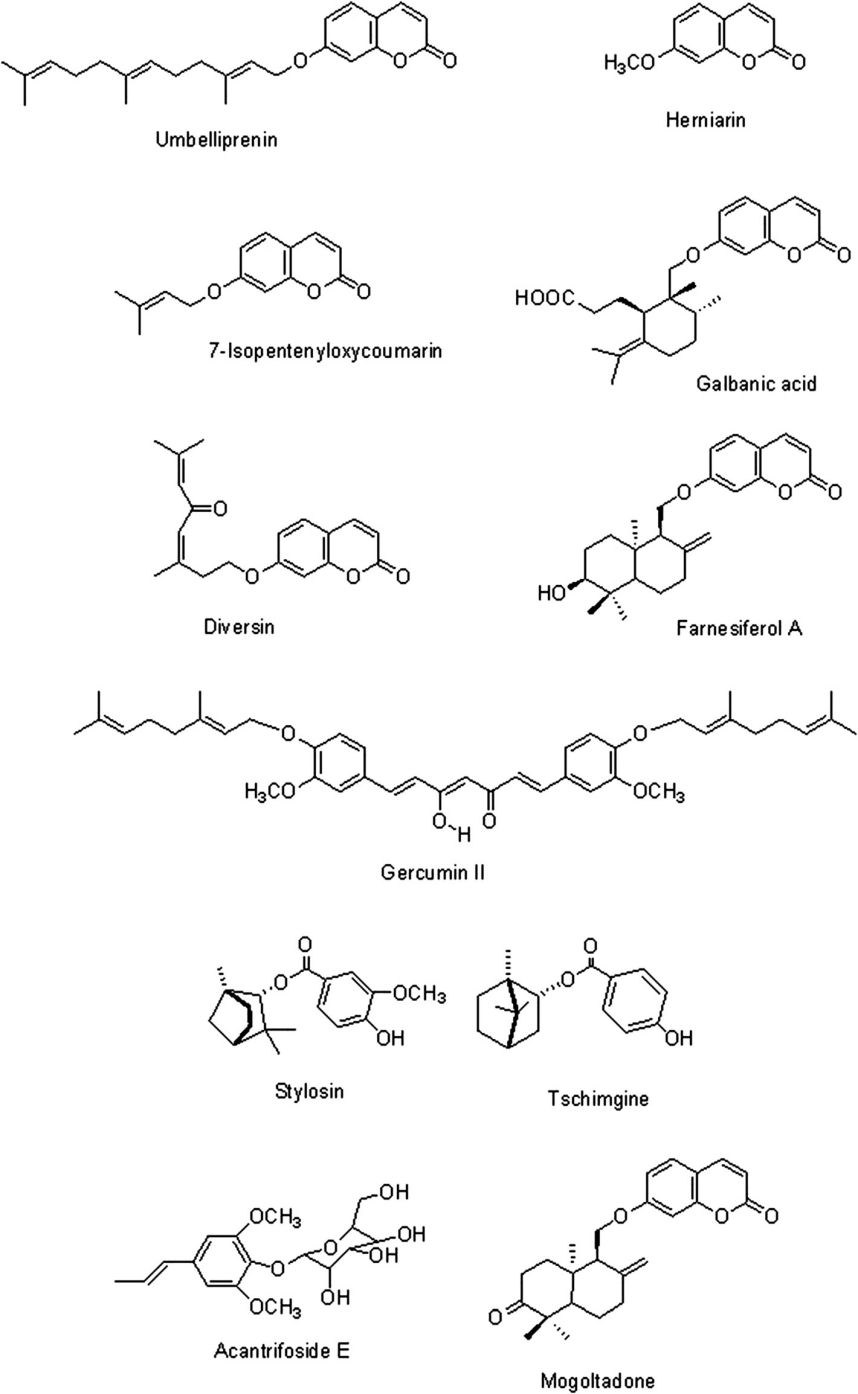
Chemical structure of tested compounds.

Cytotoxic activity of the phytochemicals was studied by means of a colorimetric microculture assay (MTT assay) in three human cancer cell lines representing different tumor entities: ovarian carcinoma (CH1), lung cancer (A549) and melanoma (SK-MEL-28) yielding IC_50_ values mostly in the micromolar range. IC_50_ values are listed in Table 1, and concentration-effect curves are depicted in Figure 2. One of the cell lines is sensitive to cisplatin (CH1), while the other two (A549, SK-MEL-28) are intrinsically resistant to cisplatin.

**Table 1:** Cytotoxicity of tested phytochemicals on three human cancer cell lines

**Compound**	**IC**_**50 **_**[μM]**^**a**^		
	**A549**	**SK-MEL-28**	**CH1**
**Conferone**	38.8 ± 7.0	63.8 ± 30.0	7.79 ± 3.49
**Farnesiferol A**	53.7 ± 5.1	213 ± 17	25.7 ± 1.2
**Stylosin**	79.0 ± 5.9	44.3 ± 5.4	24.1 ± 4.3
**Diversin**	> 250	> 250	> 62,5
**Herniarin**	> 250	> 250	> 62,5
**Galbanic Acid**	89.1 ± 4.4	76.1 ± 5.4	46.3 ± 2.8
**Mogoltadone**	86.0 ± 18.2	159 ± 29	21.0 ± 3.1
**7**-**Isopentenyloxycoumarin**	> 250	> 250	> 62.5
**Tschimgine**	46.6 ± 1.8	44.6 ± 0.7	38.4 ± 0.8
**Acantrifoside E**	> 250	> 250	> 62.5
**Umbelliprenin**	133 ± 6	58.1 ± 15.2	37.2 ± 2.7
**Gercumin II**	> 250	> 250	> 62.5
**Cisplatin**	1.3 ± 0.3	2.2 ± 0.05	0.2 ± 0.03

^a^ 50% inhibitory concentrations in *CH1*, A549 and *SK*-*MEL*-*28* cells in the *MTT* assay. Values are means ± standard deviations obtained from at least three independent experiments.

To our knowledge, data regarding cytotoxic activity of the compounds tested herein have been very few and for some of them lacking. All tested compounds displayed cytotoxic activity against the tumor cell lines. However, the obtained IC_50_ values for all evaluated compounds were markedly higher than that of the reference standard, cisplatin. In addition, no obvious structure-activity relationship was found for the tested compounds. Overall, conferone exhibited the highest activity among the tested compounds against A549 and CH1 cell lines. In previous investigations, Barthomeuf and colleagues demonstrated that conferone enhances the cytotoxic activity of vinblastine in MDCK-MDR1 cells. This activity was shown to be due to the potent inhibition of P-glycoprotein by this phytochemical and consequent reversal of multidrug resistance [15]. The same effect of conferone has also been reported for vincristine against 5637 cell line [16]. However, in both of these studies, treatment of cell lines with conferone alone did not result in any considerable cytotoxic activity. As for the SK-MEL-28 cell line, the highest activity was observed from stylosin and tschimgine. Previous investigations regarding the cytotoxic properties of these phytochemicals have been very scant. Findings of two previous studies have implied the anti-tumor activity of stylosin and tschimgine against 5637 and MCF-7 cell lines, respectively [17,18]. The cytotoxic activities of these monoterpenes could be attributed, at least in part, to their phytoestrogenic properties as phytoestrogens have been postulated to possess cytotoxic effects [19,20]. As for the umbelliprenin, previous investigations have shown that this phytochemical possesses *in vitro* and *in vivo* chemopreventive as well as *in vitro* anti-tumor properties [21-23]. The anti-tumor activity of this agent has been documented to be mediated through cell cycle arrest at G1 phase and induction of caspase-dependent apoptosis [21]. Moreover, umbelliprenin has been reported to inhibit matrix metalloproteinases and therefore might be effective against tumor invasion, metastasis and angiogenesis [24]. Nevertheless, it appears that direct cytotoxic activity of umbelliprenin varies based on the specificity of this phytochemical for different cell lines. Whilst the effects of umbelliprenin was found to be superior to cisplatin in M4Beu cells, no such an effect was found in other cell lines including DLD1, MCF7, PA1, PC3 and A549 [24]. Galbanic acid has also been reported to inhibit VEGF-induced proliferation, migration and angiogenesis, thereby possessing anti-tumor activity [25]. Finally, a recent report by Hanafi-Bojd et al. has indicated the inhibitory activity of galbanic acid and farnesiferol A against P-glycoprotein, thereby posing their potential efficacy in the treatment of multidrug resistant tumors [26]. In accordance with our findings, a recent study by Iranshahi *et al*. did not found any significant activity of isolated sesquiterpene coumarins from *F*. *gummosa* against M14, MCF-7, T98G, A549, Saos-2, FRO, and U937 cell lines. The only exception was the cytotoxic effect of feselol against the U937 cell line [13]. Another miscellaneous compound that was investigated in the present study was gercumin II, a novel synthetic derivative of curcumin. There has been a great deal of previous work indicating the anti-carcinogenic and chemopreventive activity of curcumin against a wide variety of cancers [27-34]. It appears that the cytotoxic activity of curcumin is mediated by multiple mechanisms including anti-inflammatory, free radical scavenging, anti-genotoxic, anti-angiogenic and anti-metastatic properties [33]. Gercumin II is a synthetic germacryl derivative of curcumin which has 10 C prenyl moieties substituted on the phenolic hydroxyl groups of curcumin. However, this prenylation was not found to enhance the cytotoxic activity of curcumin as gercumin II was almost inactive against all tested cell lines. Nevertheless, the impact of curcumin prenylation on other related biological activities of curcumin such as chemopreventive, chemomodulatory and anti-angiogenic activities remain to be explored.

## Conclusions

In summary, the findings of the present investigation did not support a potent cytotoxic activity of the tested phytochemicals against CH1, A549 and SK-MEL-28 cell lines. Lack of remarkable anti-tumor activity of the tested compounds in the present study, which is in contrast with some previous findings, might be attributed to the possible differential effects of these phytochemicals against different cell lines. Besides, since antioxidants have a dual role in fighting cancer [35], it remains to be determined if the antioxidant properties (in terms of altering intracellular content of reactive oxygen species) of compounds tested in this study differ among cancerous cell lines, and also there is any association between the observed cytotoxic properties and degree of antioxidant activity for each compound. Another issue that merits further investigation is the potential cytotoxic properties of crude *Ferula* extracts as these extracts are known to possess antioxidant properties which is mainly due to their phenolic constituents [36]. Finally, it appears that the tested agents might be more effective in term of cancer therapy as chemopreventive or chemosensitizers, as previously shown for some.

## Additional files

NMR spectral data of tested compounds are available as supporting information.

## Competing interests

The authors have no conflict of interests to declare.

## Authors’ contributions

AS and SMV conceived the study and designed the experiments. MI and SMV performed the experimental work including phytochemical and cell culture investigations, respectively. AS and SMV were involved in data interpretation and drafting the manuscript. All authors read and approved the final manuscript.

## Supplementary Material

Additional file 1 ^**1**^**H-NMR spectrum of umbelliprenin.** Click here for file

Additional file 2 ^**1**^**H-NMR spectrum of 7-isopentenyloxycoumarin.** Click here for file

Additional file 3 ^**1**^**H-NMR spectrum of herniarin.** Click here for file

Additional file 4 ^**1**^**H-NMR spectrum of stylosin.** Part A. Click here for file

Additional file 5 ^**1**^**H-NMR spectrum of stylosin.** Part B. Click here for file

Additional file 6 ^**1**^**H-NMR spectrum of stylosin.** Part C. Click here for file

Additional file 7 ^**1**^**H-NMR spectrum of tschimgine.** Part A. Click here for file

Additional file 8 ^**1**^**H-NMR spectrum of tschimgine.** Part B. Click here for file

Additional file 9 ^**1**^**H-NMR spectrum of galbanic acid.** Click here for file

Additional file 10 ^**1**^**H-NMR spectrum of farnesiferol A.** Click here for file

Additional file 11 ^**1**^**H-NMR spectrum of diversin.** Click here for file

Additional file 12 ^**1**^**H-NMR spectrum of conferone.** Click here for file

Additional file 13 **H-NMR spectrum of acantrifoside E.** Part A. Click here for file

Additional file 14 ^**1**^**H-NMR spectrum of acantrifoside E.** Part B. Click here for file

Additional file 15 ^**1**^**H-NMR spectrum of acantrifoside E.** Part C. Click here for file

Additional file 16 ^**1**^**H-NMR spectrum of mogoltadone.** Click here for file

Additional file 17 ^**1**^**H-NMR spectrum of gercumin II.** Click here for file
