# Supplementary figures and images for: Cytotoxic activities of phytochemicals from Ferula species
Source: Daru. 2013 May 23;21(1):39. doi: 10.1186/2008-2231-21-39 (PMC3671137; doi:10.1186/2008-2231-21-39)

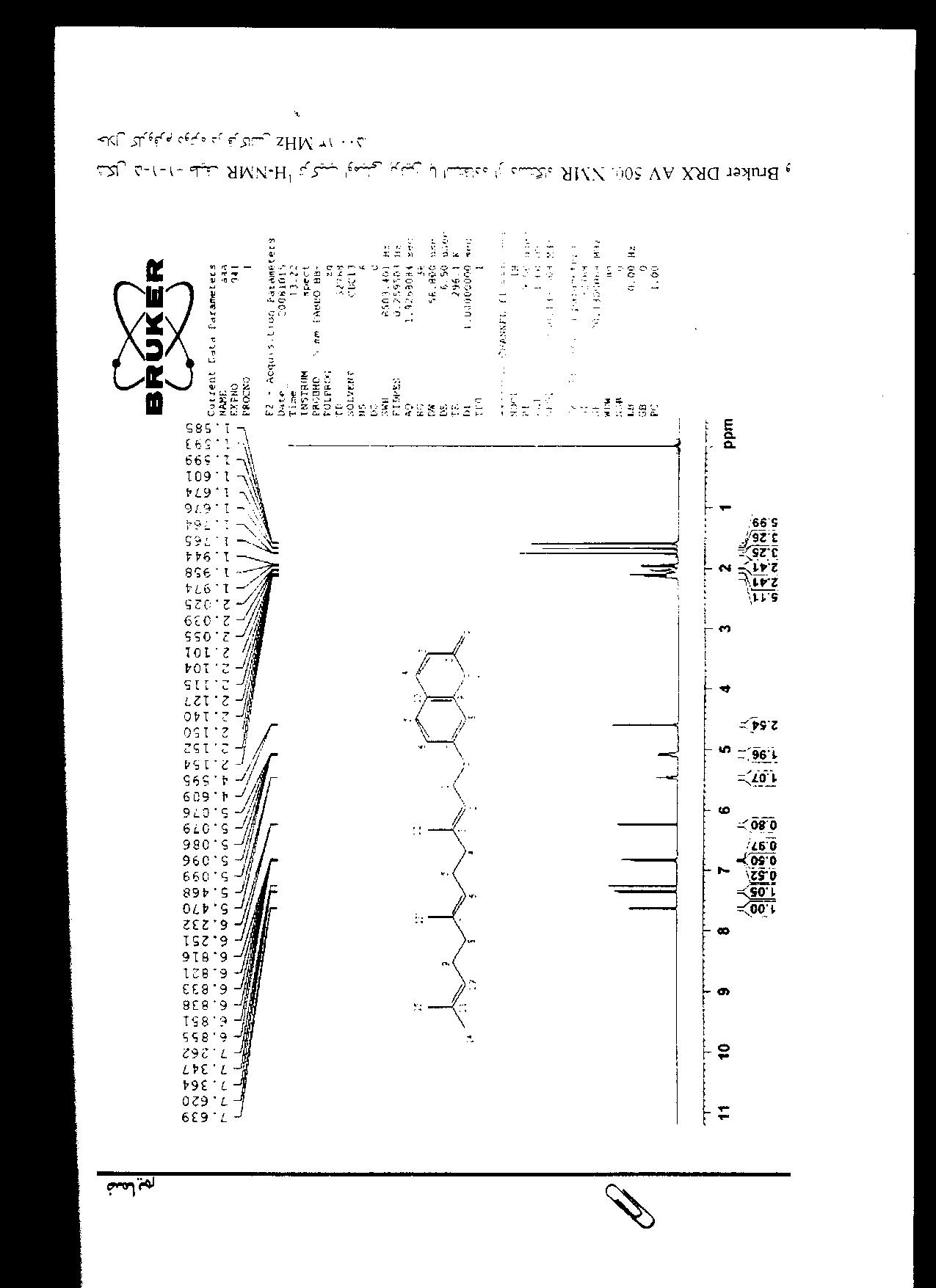

Supplement: Additional file 1 — 1H-NMR spectrum of umbelliprenin. [file 2008-2231-21-39-S1.jpeg]

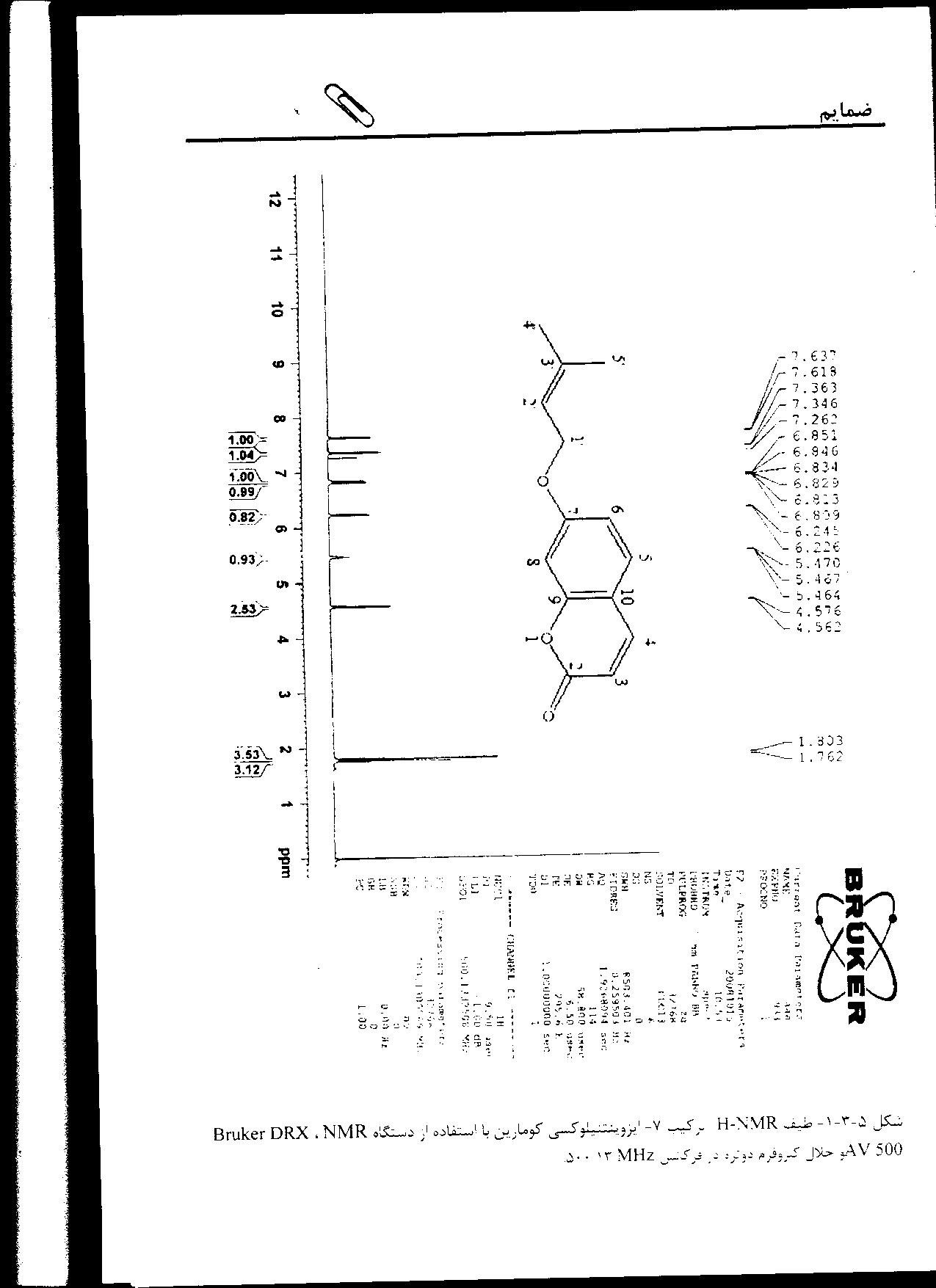

Supplement: Additional file 2 — 1H-NMR spectrum of 7-isopentenyloxycoumarin. [file 2008-2231-21-39-S2.jpeg]

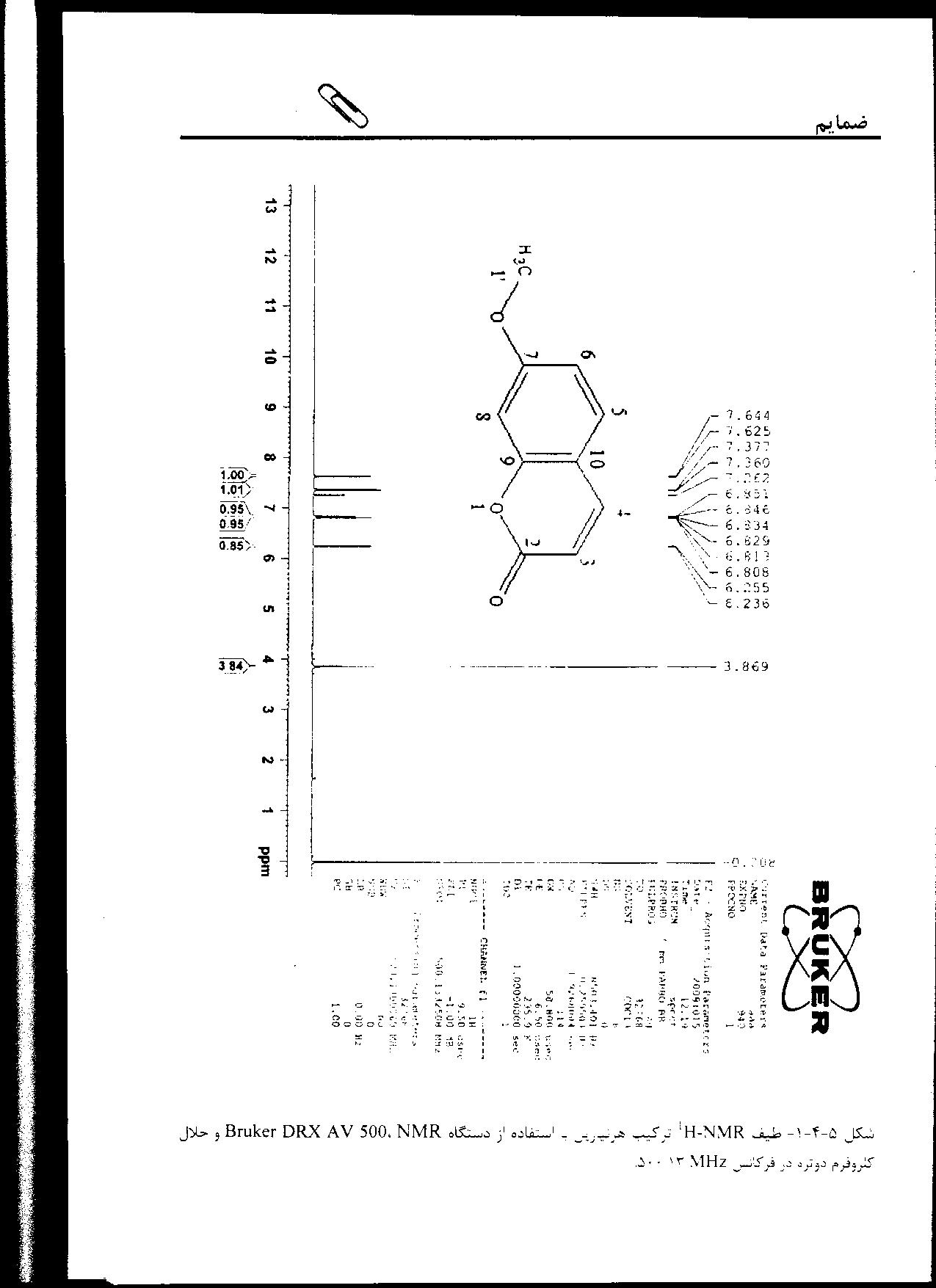

Supplement: Additional file 3 — 1H-NMR spectrum of herniarin. [file 2008-2231-21-39-S3.jpeg]

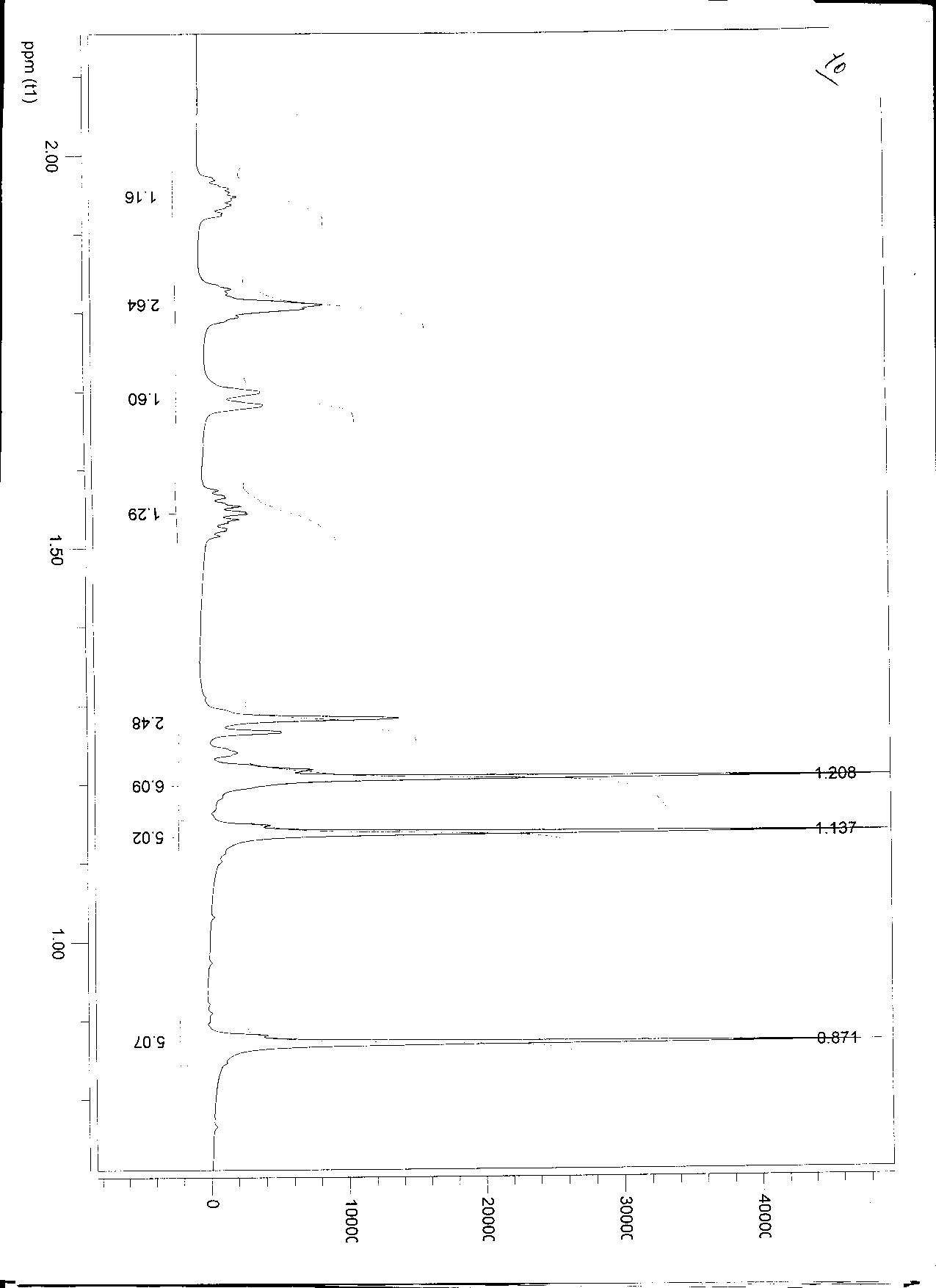

Supplement: Additional file 4 — 1H-NMR spectrum of stylosin. Part A. [file 2008-2231-21-39-S4.jpeg]

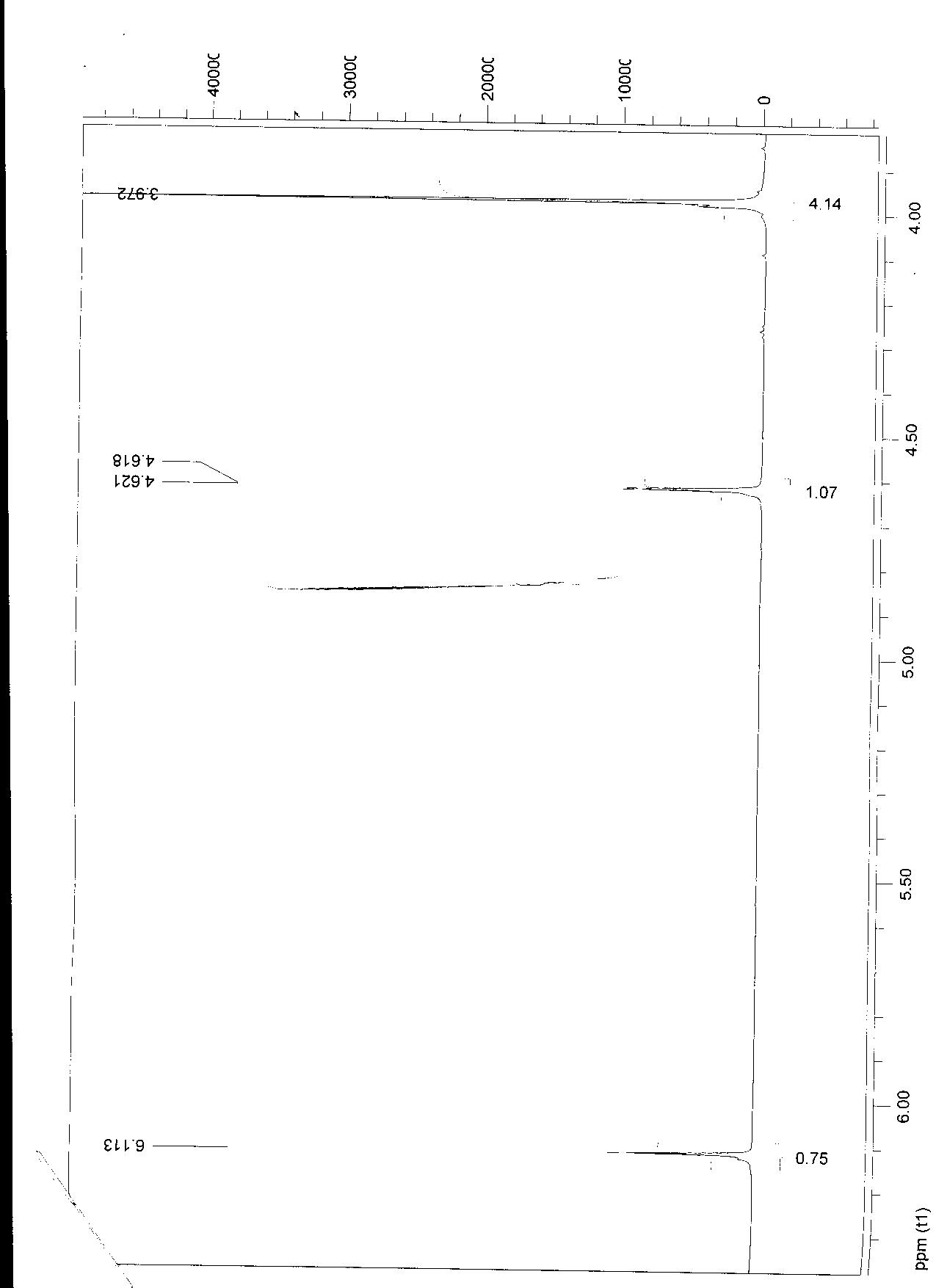

Supplement: Additional file 5 — 1H-NMR spectrum of stylosin. Part B. [file 2008-2231-21-39-S5.jpeg]

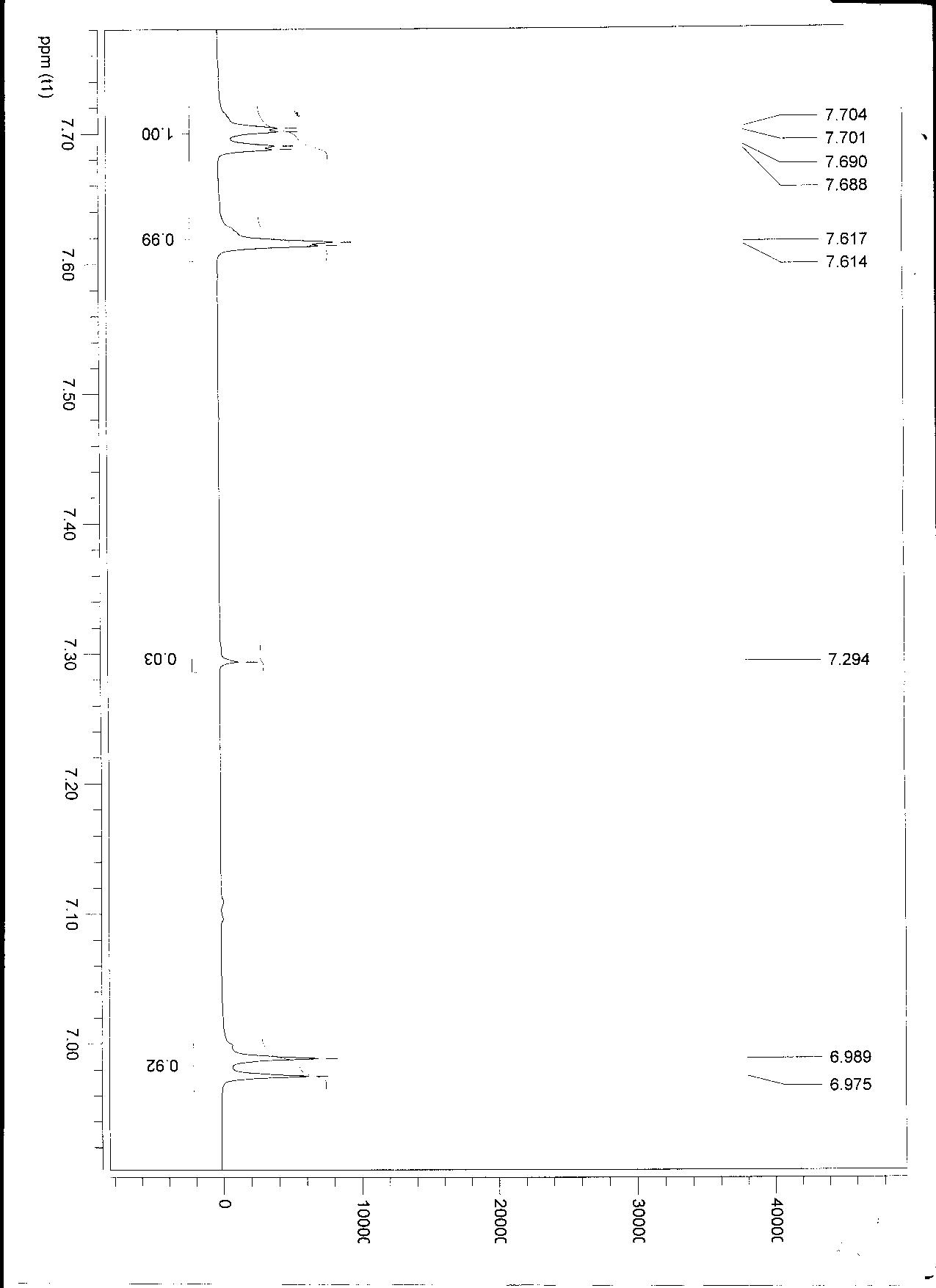

Supplement: Additional file 6 — 1H-NMR spectrum of stylosin. Part C. [file 2008-2231-21-39-S6.jpeg]

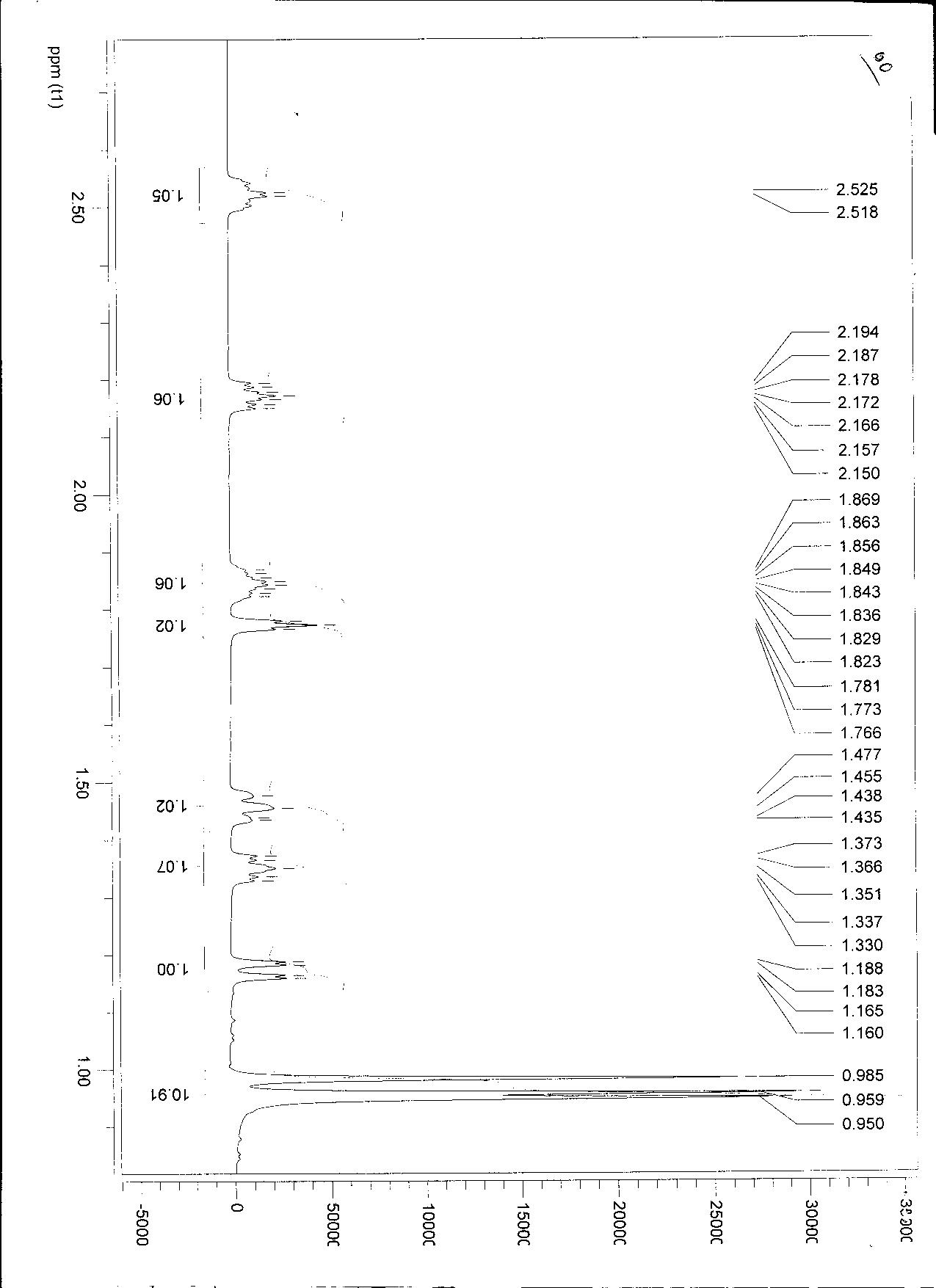

Supplement: Additional file 7 — 1H-NMR spectrum of tschimgine. Part A. [file 2008-2231-21-39-S7.jpeg]

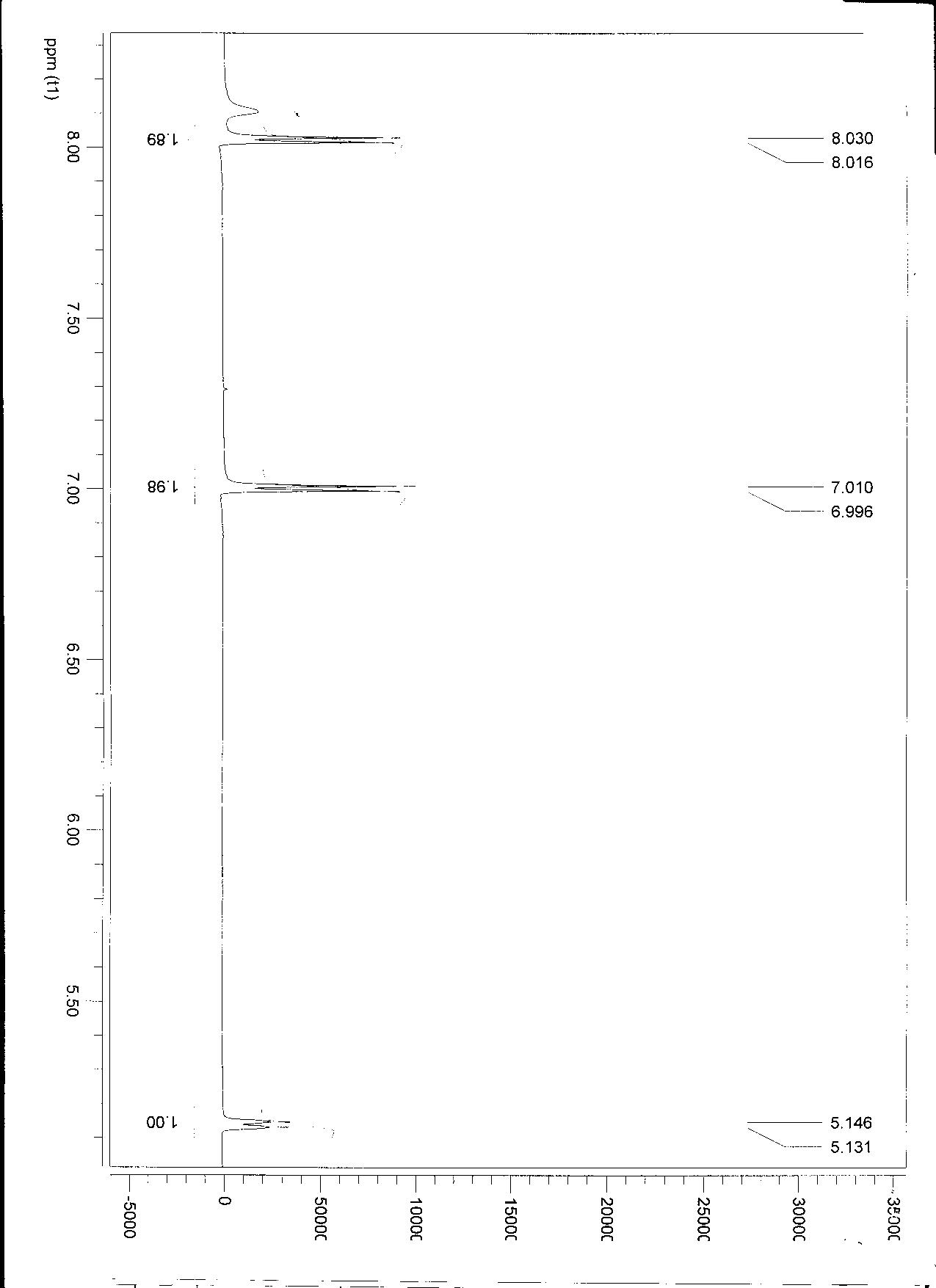

Supplement: Additional file 8 — 1H-NMR spectrum of tschimgine. Part B. [file 2008-2231-21-39-S8.jpeg]

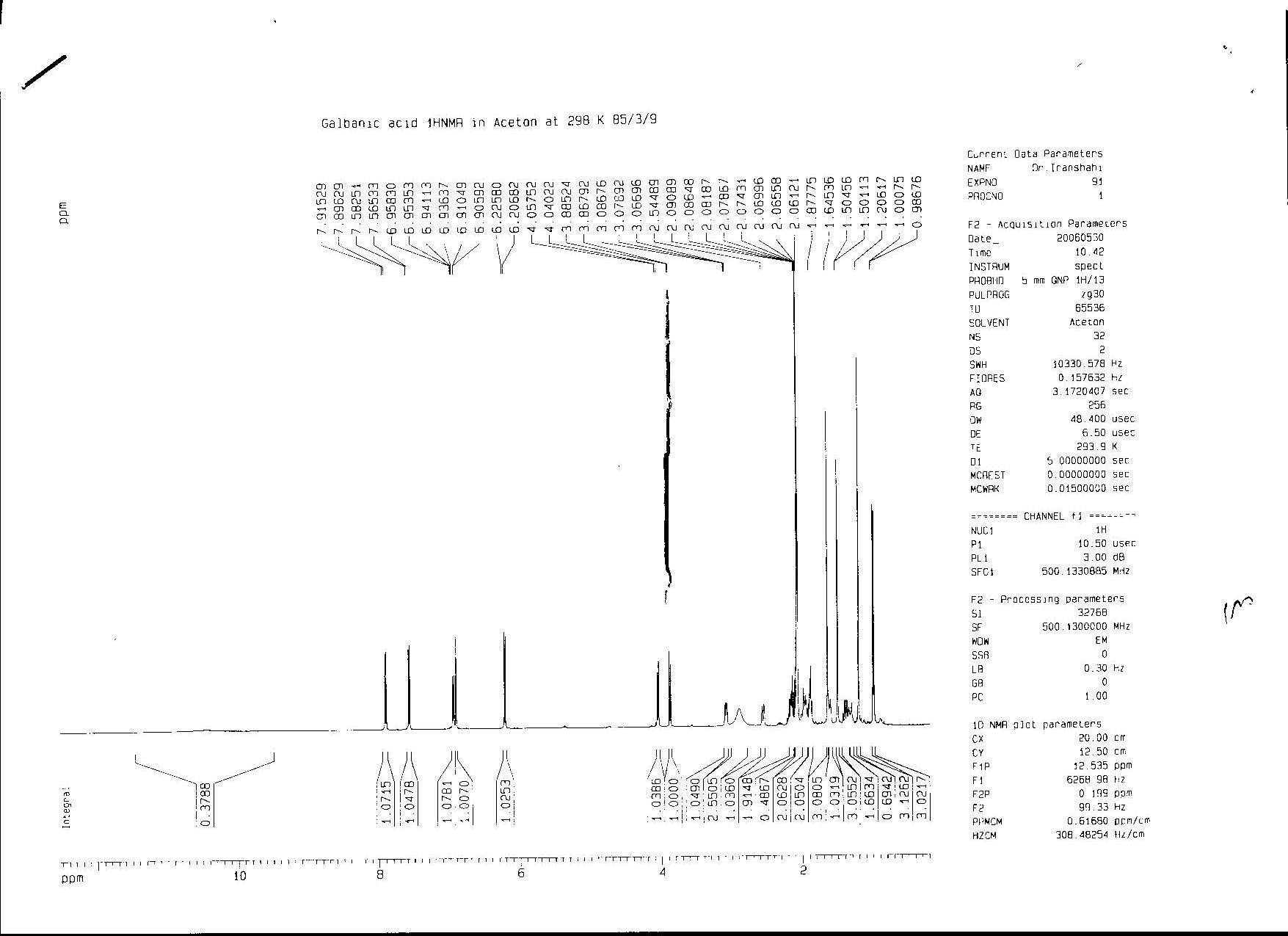

Supplement: Additional file 9 — 1H-NMR spectrum of galbanic acid. [file 2008-2231-21-39-S9.jpeg]

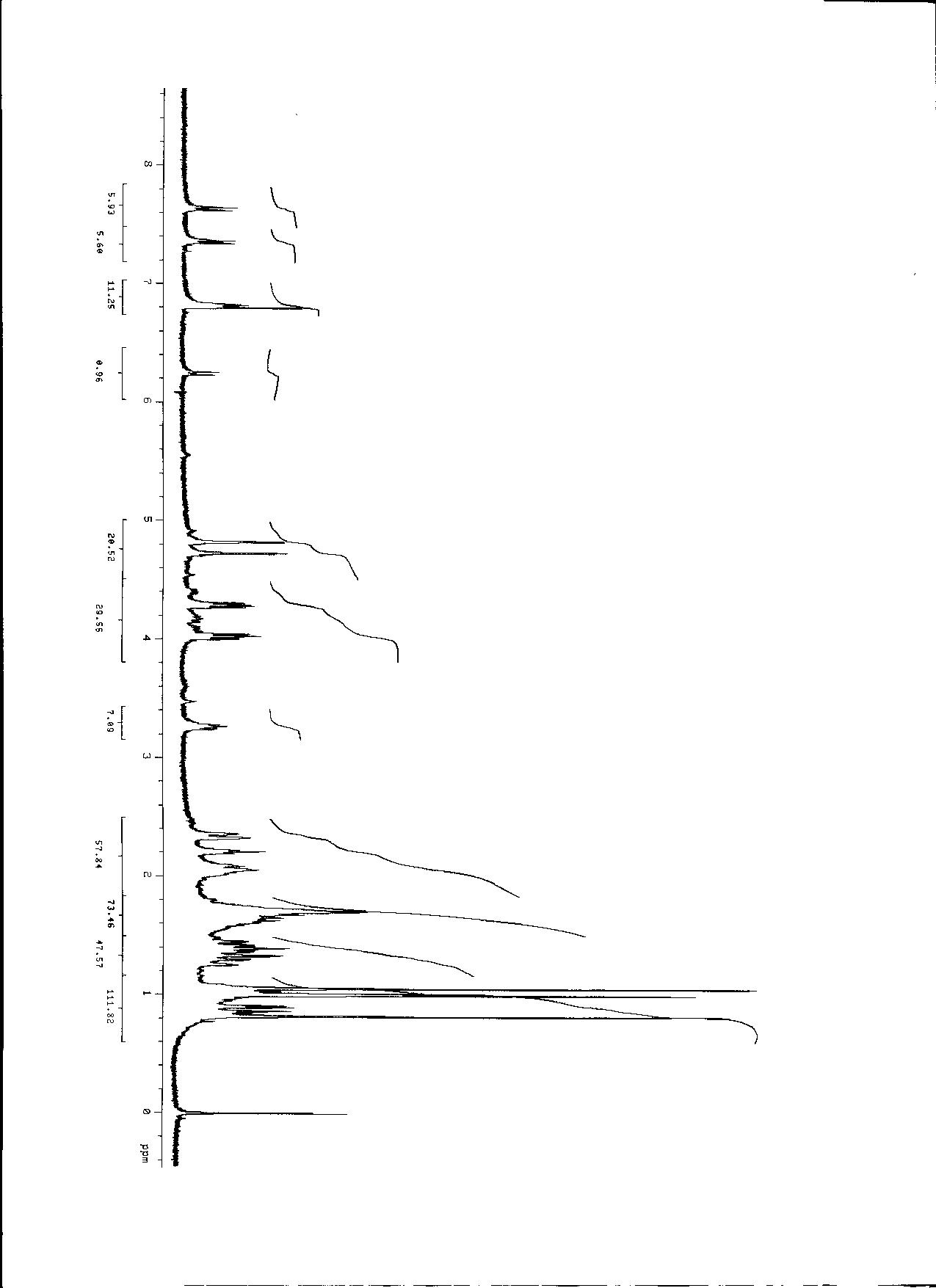

Supplement: Additional file 10 — 1H-NMR spectrum of farnesiferol A. [file 2008-2231-21-39-S10.jpeg]

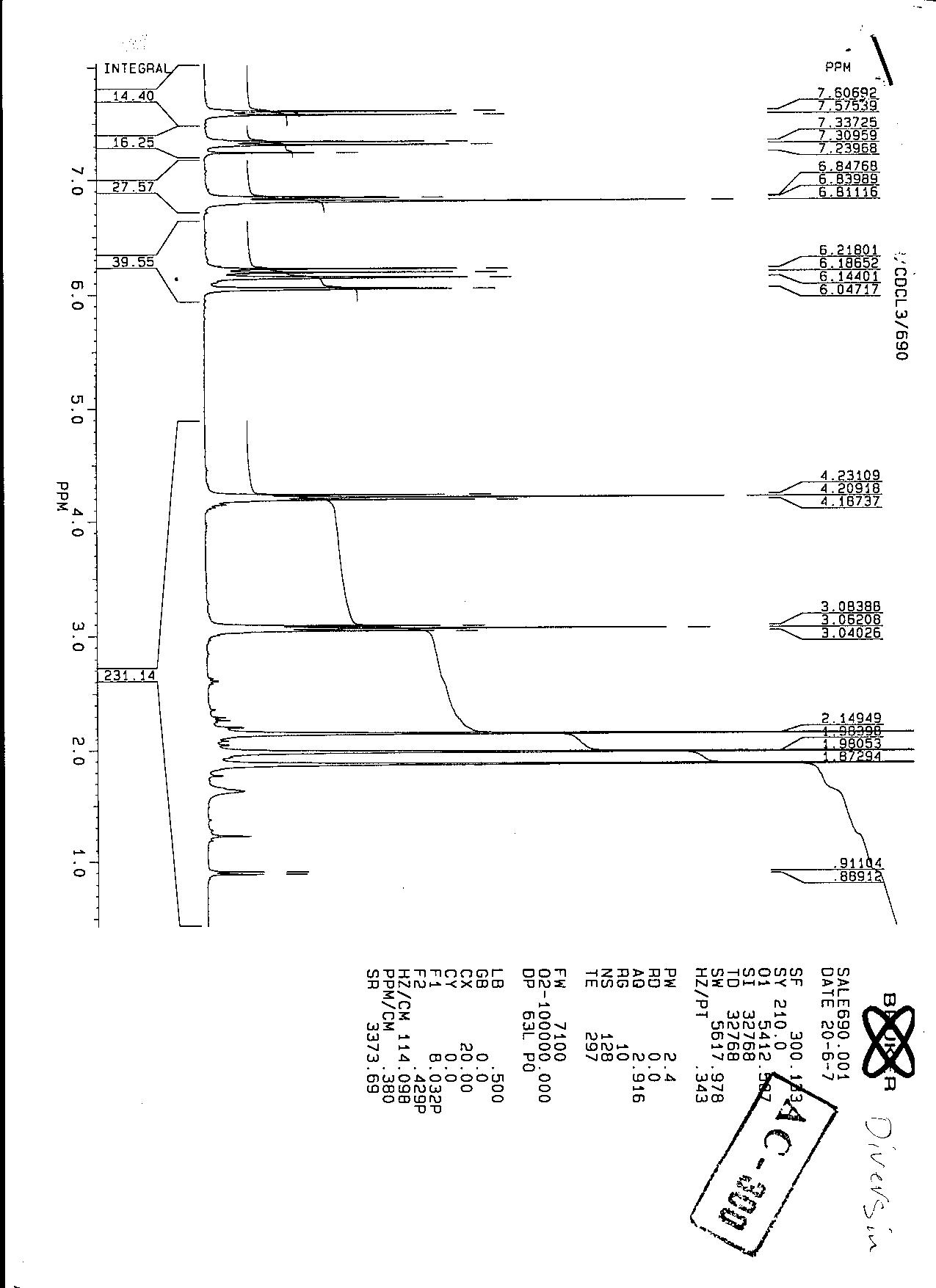

Supplement: Additional file 11 — 1H-NMR spectrum of diversin. [file 2008-2231-21-39-S11.jpeg]

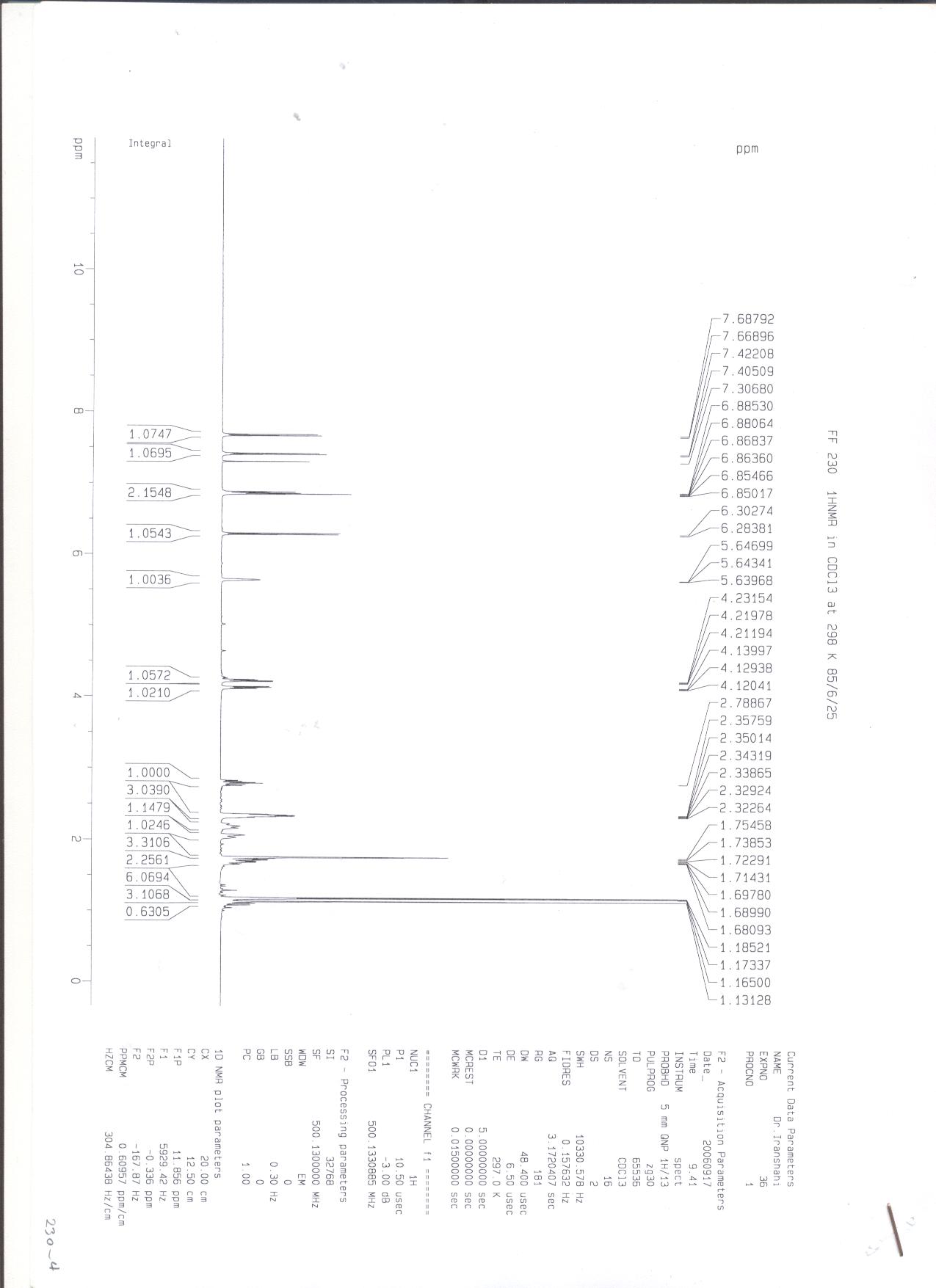

Supplement: Additional file 12 — 1H-NMR spectrum of conferone. [file 2008-2231-21-39-S12.jpeg]

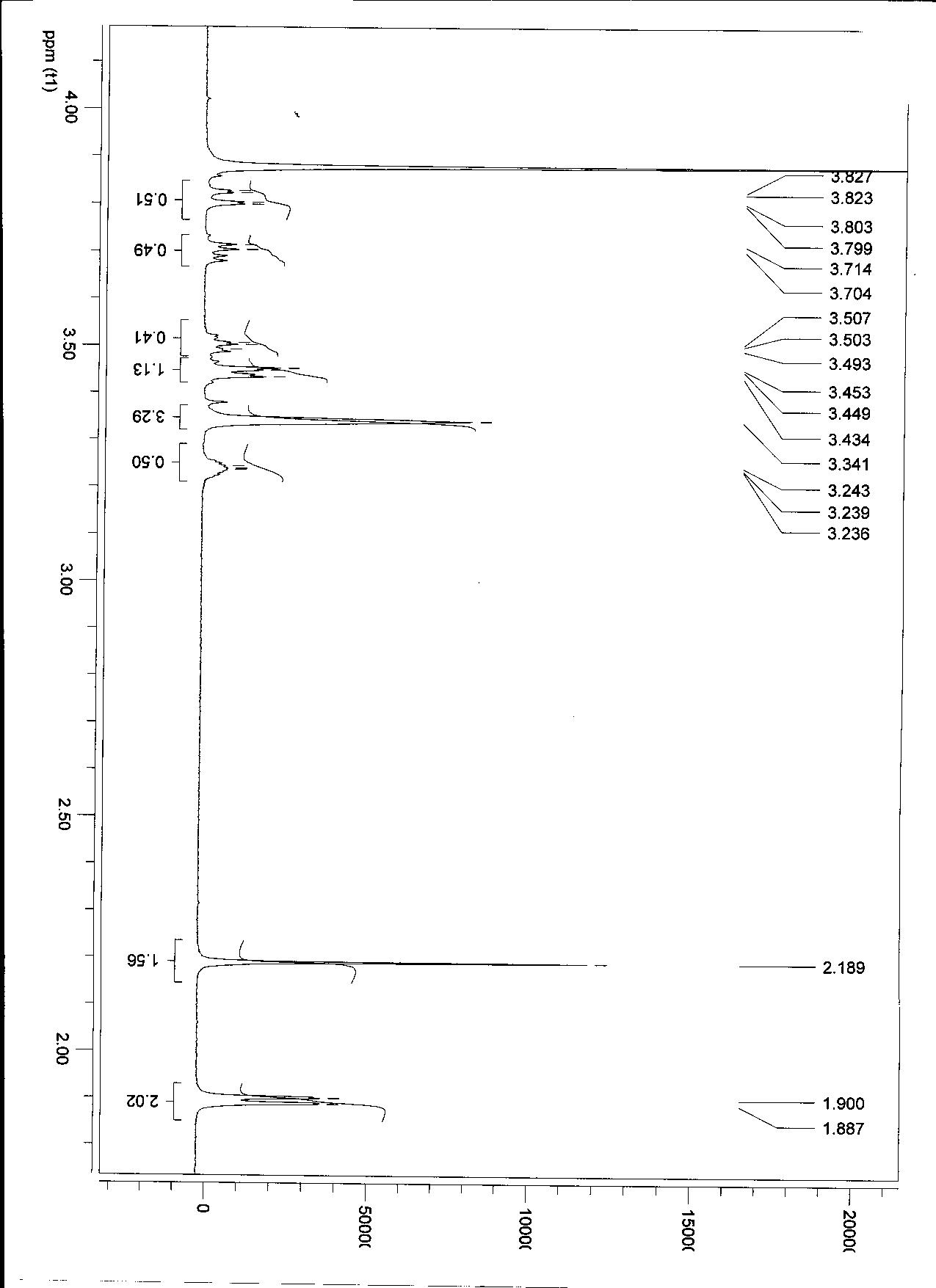

Supplement: Additional file 13 — H-NMR spectrum of acantrifoside E. Part A. [file 2008-2231-21-39-S13.jpeg]

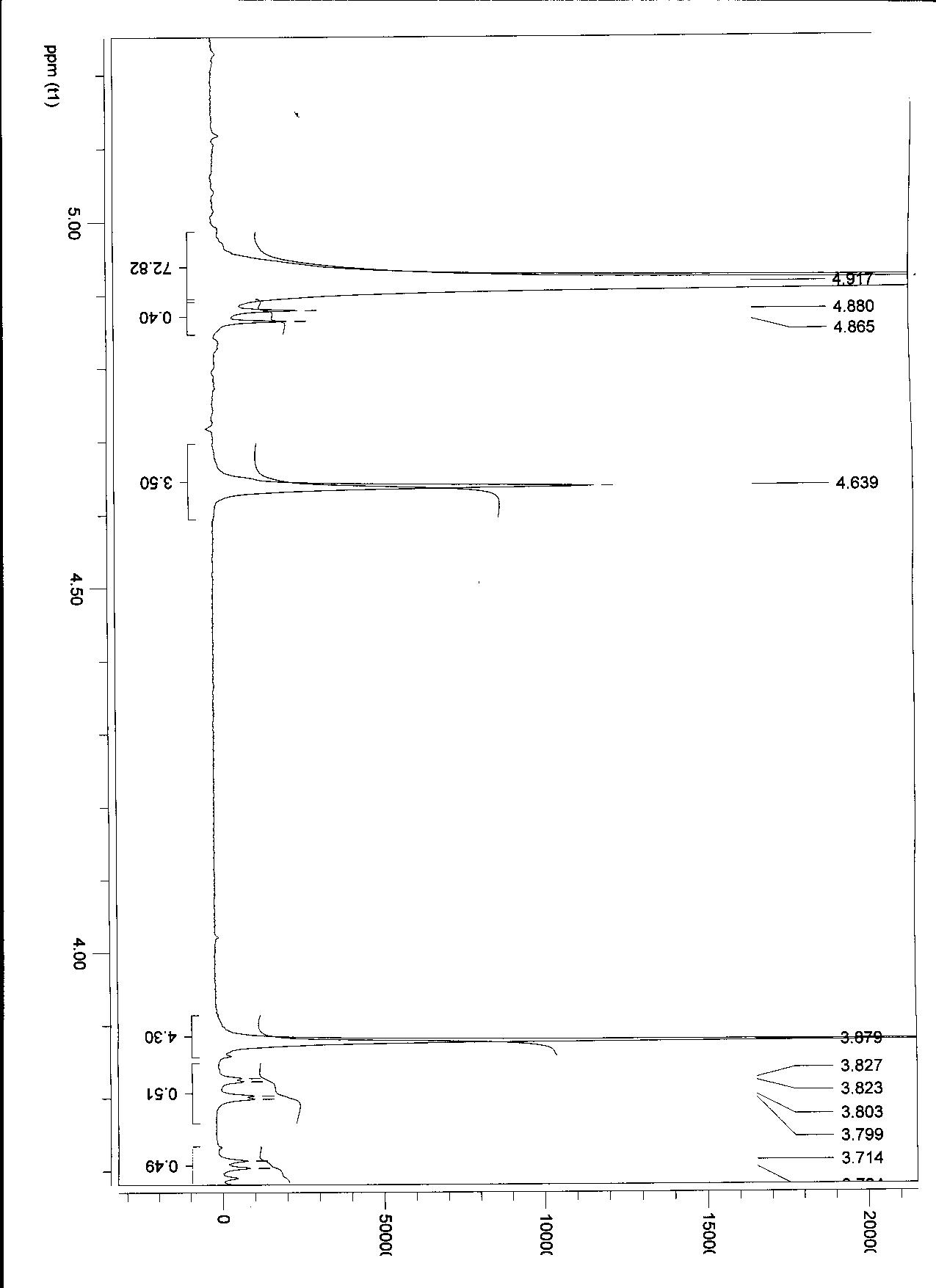

Supplement: Additional file 14 — 1H-NMR spectrum of acantrifoside E. Part B. [file 2008-2231-21-39-S14.jpeg]

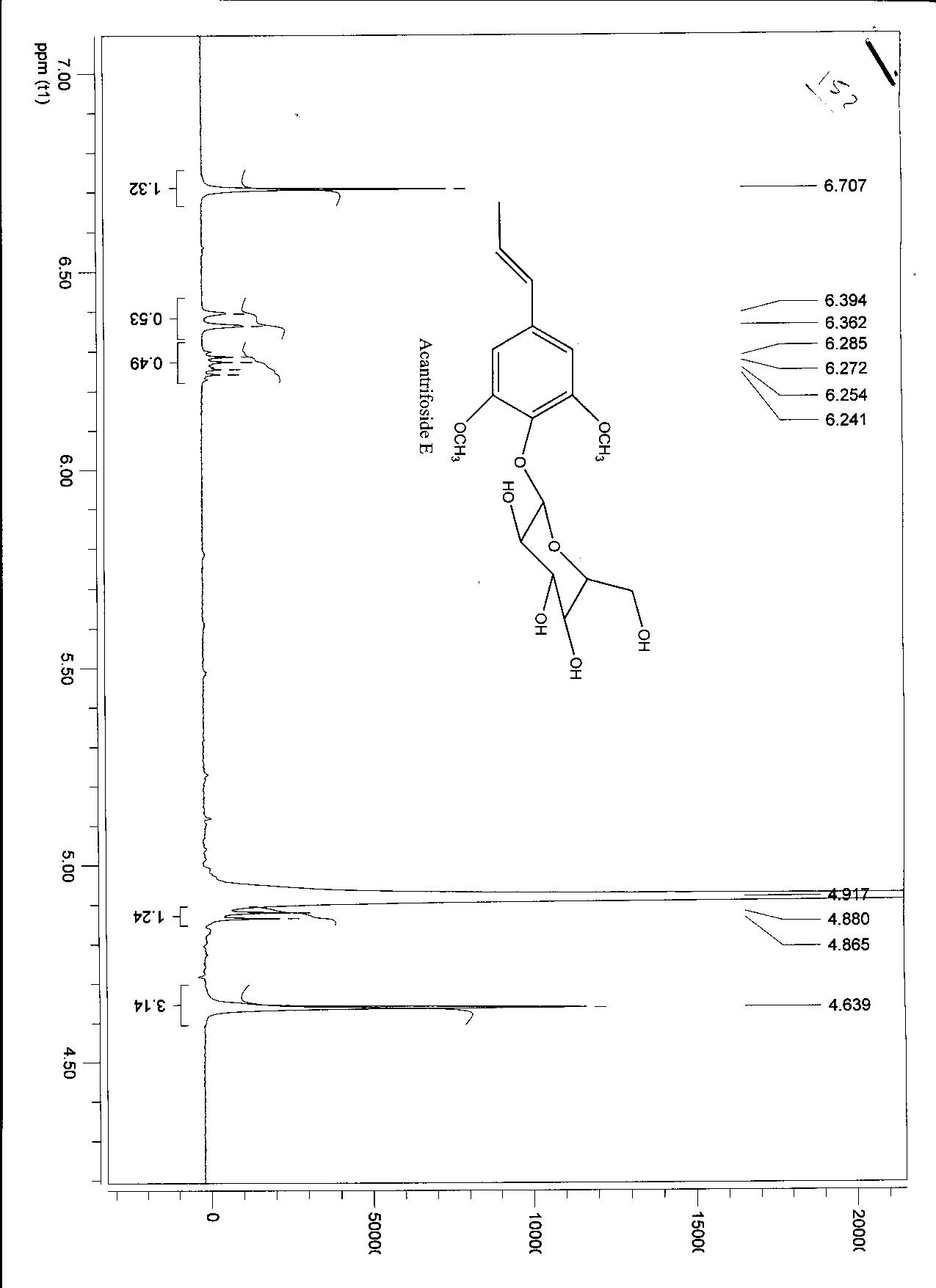

Supplement: Additional file 15 — 1H-NMR spectrum of acantrifoside E. Part C. [file 2008-2231-21-39-S15.jpeg]

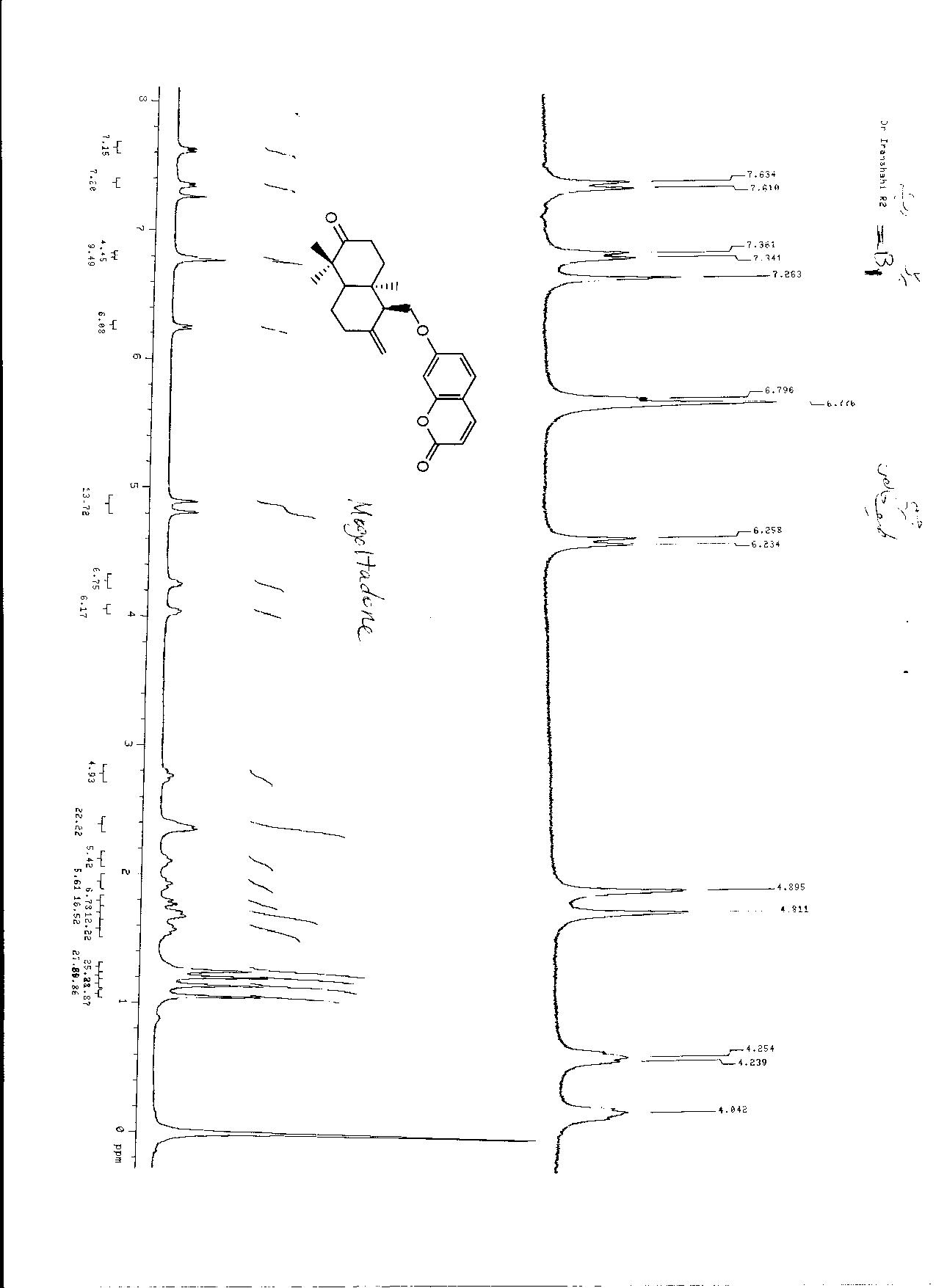

Supplement: Additional file 16 — 1H-NMR spectrum of mogoltadone. [file 2008-2231-21-39-S16.jpeg]

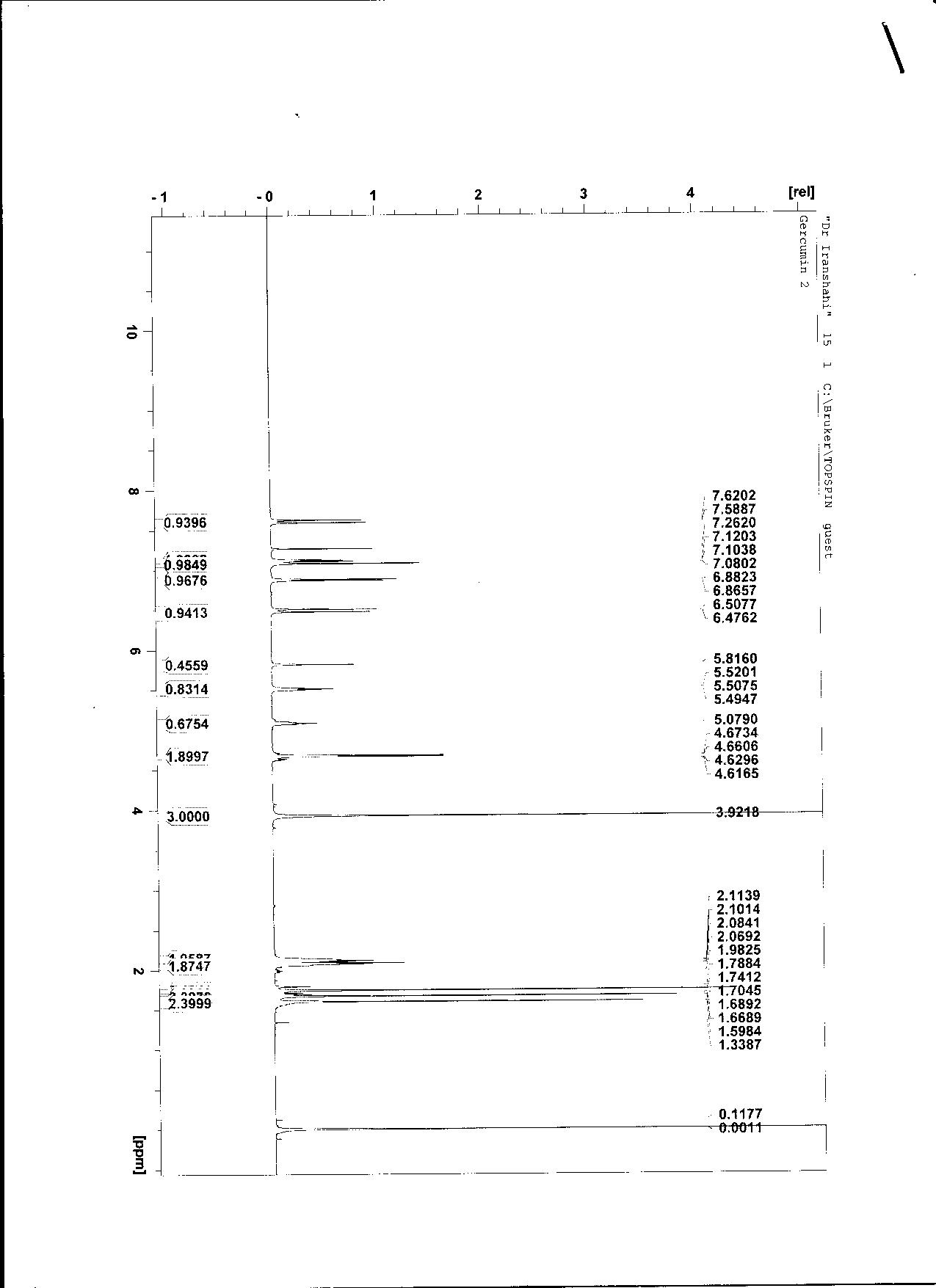

Supplement: Additional file 17 — 1H-NMR spectrum of gercumin II. [file 2008-2231-21-39-S17.jpeg]
